# Neuropharmacological and Antipyretic Potentials of *Streblus asper* Leaves: Integrated In Vivo and In Silico Approaches

**DOI:** 10.1155/tswj/7661411

**Published:** 2026-04-13

**Authors:** Md. Hossain Rasel, Md. Jahirul Islam Mamun, Salma Sultana Sumi, Md. Nahid Hasan, Minhajur Rahman, S. M. Naim Uddin

**Affiliations:** ^1^ Department of Pharmacy, University of Chittagong, Chittagong, 4331, Bangladesh, cu.ac.bd; ^2^ Department of Chemistry, University of Chittagong, Chittagong, 4331, Bangladesh, cu.ac.bd; ^3^ Department of Botany, University of Chittagong, Chittagong, 4331, Bangladesh, cu.ac.bd

**Keywords:** antidepressant, antipyretic, anxiolytic, in silico, sedative, *Streblus asper*

## Abstract

*Streblus asper* (Moraceae) is traditionally used for neurological and febrile disorders, but its pharmacological basis remains unclear. This study evaluated the *S. asper* leaf methanolic extract (SAL‐ME) for anxiolytic, antidepressant, sedative, and antipyretic activities using Swiss albino mice and in silico docking analyses. Behavioral assays included the elevated plus maze, hole‐board, forced swim, tail suspension, hole cross, and open field tests, while brewer’s yeast‐induced pyrexia was used to assess antipyretic activity. SAL‐ME (200 and 400 mg/kg) produced dose‐dependent effects, significantly reducing immobility time (*p* < 0.001), increasing open‐arm exploration (*p* < 0.01), and suppressing locomotor activity, indicating antidepressant, anxiolytic, and sedative actions. A significant antipyretic effect was observed at 400 mg/kg, with a marked reduction in rectal temperature within 3 h posttreatment (*p* < 0.01). Molecular docking analysis revealed notable binding affinities of octadecanoic acid, hexadecanoic acid, D‐pinitol, α‐D‐glucopyranoside, myo‐inositol, and butanedioic acid with target proteins associated with GABAergic, serotonergic, and prostaglandin‐mediated pathways. Collectively, these findings suggest that SAL‐ME exerts dose‐dependent, multitarget pharmacological effects, supporting its potential as a phytotherapeutic candidate for CNS disorders and fever.

## 1. Introduction

Anxiety disorders are among the most common neuropsychiatric conditions, affecting nearly 20% of adults during their lifetime [1]. While mild anxiety is a normal adaptive response, persistent or severe anxiety can significantly impair quality of life. Current treatments primarily include benzodiazepine receptor agonists and selective reuptake inhibitors of serotonin, dopamine, and noradrenaline [2]. However, these drugs often produce undesirable effects such as dependence, cognitive dysfunction, and psychomotor impairment [3–5]. Similar concerns exist for depression, which affects approximately 260 million people globally [6] and frequently co‐occurs with anxiety [7, 8]. Antidepressants like SSRIs, though effective, are associated with adverse effects that compromise adherence [9, 10].

Insomnia, another common disorder, contributes to cardiovascular and metabolic risks and often arises from stress, anxiety, or depression [11, 12]. Pharmacological treatments, including benzodiazepines and nonbenzodiazepine hypnotics, carry risks of tolerance, dependence, and residual sedation [13, 14]. Likewise, fever—a frequent symptom of infection and inflammation—is typically managed using NSAIDs and acetaminophen, but their misuse can cause hepatotoxicity, nephrotoxicity, and allergic reactions [15, 16]. These limitations have stimulated interest in safe, plant‐based alternatives for CNS disorders and fever [17, 18].

*Streblus asper* Lour*.* (family: Moraceae) is a shrub commonly found across South and Southeast Asia and has been extensively used in traditional medicine for the management of fever, anxiety, pain, and various infections [19, 20]. This plant exhibits a wide range of pharmacological applications. For example, the bark extract is traditionally employed to alleviate fever, treat dysentery, relieve toothache, and manage gingivitis [21]. The leaves have demonstrated insecticidal properties, particularly against mosquito larvae [22], while the twigs are commonly used as natural “toothbrushes” to strengthen teeth and gums [23]. Additionally, the roots have been applied topically to treat chronic ulcers and sinus infections and are also used as a local remedy for snake bites. The milky latex of the plant possesses antiseptic and astringent properties and is applied to care for chapped hands and cracked heels [24]. Despite these diverse ethnomedicinal uses, scientific investigations into the neuropharmacological and antipyretic potential of its leaves are still limited, highlighting the need for further research in this area.

This study aimed to evaluate the anxiolytic, antidepressant, sedative, and antipyretic activities of the *S. asper* leaf methanolic extract (SAL‐ME) through in vivo behavioral models and to investigate underlying mechanisms via in silico molecular docking and ADMET prediction. By combining experimental and computational approaches, this work provides scientific validation for the traditional uses of *S. asper* and identifies potential bioactive phytochemicals for future drug development.

## 2. Materials and Methods

### 2.1. Plant Collection and Identification

In March 2024, *S*. *asper* leaves were collected from Mirsharai, Chattogram, Bangladesh. Professor Dr. Shaikh Bokhtear Uddin of the Department of Botany, University of Chittagong, Bangladesh, verified the samples’ taxonomy. A voucher specimen was placed in the University of Chittagong, Chittagong’s herbarium center after being given the accession number (4321).

### 2.2. Preparation of the Plant Extract

The leaves of *S. asper* were collected, washed, and separated from undesired plant parts. The air‐drying process lasted for a week. A coarse powder was made from the fruits using laboratory milling equipment. Cold extraction was employed in the extract preparation process. A glass container with a clean, flat bottom and about 700 g of powdered material was filled with 2.5 L of 80% methanol. The container and its contents were sealed, shaken, and stirred occasionally for 15 days to increase the extraction’s efficacy. The entire mixture was then filtered through a piece of fresh, white cotton, and finally, Whatman filter paper (Bibby RE200, Sterilin Ltd., UK) was utilized. The resulting filtrate (methanol extract) was dried using a rotary evaporator. The extract was stored at 4°C in the refrigerator before usage [25].

### 2.3. Chemicals and Reagents

The Department of Pharmacy at the University of Chittagong supplies methanol along with various other chemical reagents. Fluoxetine, diazepam, and paracetamol were collected from Square Pharmaceutical Company Ltd., Bangladesh. All other chemicals used in this experiment were of high purity and suitable for laboratory analysis.

### 2.4. Experimental Animals and Ethical Statement

The Comilla University animal research department in Comilla, Bangladesh, supplied the 22–30 g Swiss albino mice used in the study. The mice were housed in polycarbonate cages under standard circumstances (25 ± 2°C and 55%–60% humidity) with a 12‐h sunshine cycle [26]. Food and water were easily accessible to the animals utilized in this study. The animal trial was approved by the Ethical Review Committee of the Department of Pharmacy at the University of Chittagong, with approval number AERB‐FBSCU‐20240405‐(2A). Following each trial, the mice were administered sodium pentobarbital anesthesia to ensure humane treatment, and the study was conducted in strict compliance with the ARRIVE guidelines to uphold ethical standards, methodological rigor, and transparency in reporting.

### 2.5. Experimental Design

The experimental, control, and reference groups consisted of male and female Swiss albino mice, with five mice in each group. A 1% Tween 80 aqueous solution was given to the control group at a dose of 10 mL/kg body weight (BW). SAL‐ME was administered orally at doses of 200 and 400 mg/kg of BW using the oral gavage technique. For the open field test (OFT), hole board test (HBT), elevated plus maze (EPM), and hole cross test (HCT), diazepam (1 mg/kg, BW, PO) was used as the reference drug. Fluoxetine (10 mg/kg, PO) was administered to assess antidepressant effects in the tail suspension test (TST) and forced forced swimming test (FST). Diazepam, fluoxetine, and paracetamol served as references for anxiolytic, sedative, antidepressant, and antipyretic activities, respectively.

### 2.6. Animal Euthanasia

All experimental procedures involving animals were reviewed and approved by the Institutional Animal Care and Use Committee (IACUC) of the University of Chittagong and were conducted in accordance with relevant national and institutional ethical standards. Humane euthanasia was performed by intraperitoneal injection of freshly prepared sodium pentobarbital at a dose of 200 mg/kg. Animals were handled carefully and monitored continuously to assess anesthetic depth, including the absence of righting, pedal withdrawal, and corneal reflexes. Death was confirmed by the permanent cessation of both respiration and cardiac activity. To further ensure humane termination, a secondary physical method (bilateral thoracotomy) was performed following AVMA guidelines. All procedures were performed by trained personnel, and the disposal of animal remains adhered to institutional biosafety regulations.

### 2.7. Acute Oral Toxicity

Acute toxicity, which may arise after administering single or multiple doses of a substance, was assessed following OECD guidelines (up and down approach) to calculate the LD_50_ of the test samples [27]. Various concentrations of the extract SAL‐ME (100, 200, 400, 1000, 2000, and 4000 mg/kg BW) were orally administered to animals (5 mice per dose). Both sexes were observed for 1 h for any signs of toxicity or mortality. The mice were then monitored hourly for the next 5–6 h. Subsequently, the mice were closely observed over the following 2 weeks for any delayed adverse effects or behavioral changes [28, 29].

### 2.8. Anxiolytic Activity

#### 2.8.1. EPM Test

The method was followed as described by Hogg et al. [30, 31]. The apparatus consisted of a central square platform (5 × 5 cm) from which four arms extended: two open arms (35 × 5 cm) and two closed arms (30 × 5 × 15 cm). All surfaces, including the walls and floors of the arms, were constructed from wood and painted black. Following Lister’s specifications, the maze was elevated 60 cm above the ground. The selected animals were randomly assigned to six groups, each comprising five mice. Group I received a control solution (Tween‐80, 10 mg/mL, po), whereas Group II was served with the reference anxiolytic agent, diazepam (1 mg/kg, po). Groups III to IV were given SAL‐ME at doses of 200 and 400 mg/kg orally. Following treatment, each mouse was placed near the center of the EPM, oriented toward a closed arm. Sixty minutes after administration, the duration spent in both the open and closed arms was recorded [32, 33].

#### 2.8.2. HBT

The hole‐board instrument used for the anxiolytic assessment consisted of a wooden chamber measuring 40 × 40 × 25 cm^3^, featuring 16 evenly spaced holes, each with a diameter of 3 cm. The board was elevated 25 cm above the floor to enable the mice to explore the holes by dipping their heads. Mice weighing between 20 and 25 g were randomly divided into four groups: control, standard, and test (200 and 400 mg/kg). The test groups received the extract orally (via oral gavage) at doses of 200 and 400 mg/kg, 60 min before testing. The control group was administered distilled water (10 mL/kg, po), while the conventional reference group received diazepam (1 mg/kg, po). Each mouse was individually placed in a corner of the apparatus and allowed to explore freely. The number of head dips—defined as the insertion of the snout into a hole—was recorded over a 5‐min observation period [34–36].

### 2.9. Antidepressant Activity

#### 2.9.1. FST

FST is still one of the most popular methods for antidepressant screening among all animal models [37]. Treatments were administered to each group as per the experimental protocol. Sixty minutes posttreatment, each mouse was individually put in a transparent glass container (25 × 15 × 25 cm^3^) filled with water maintained at a depth of 15 cm and a temperature of approximately 25° ± 1°C. Animals were randomly assigned to treatment groups, and observers scoring immobility were blinded to treatment conditions. Each trial lasted 6 min, during which the first 2 min were allocated for acclimatization. The immobility duration was recorded during the final 4 min of the test period [38].

#### 2.9.2. TST

With the aid of adhesive tape, the animals were suspended by their tails on a plastic rope that was 75 cm above the ground. Eight minutes were spent observing the length of immobility. The final 6 min of the observation period were used to record the length of immobility. Only when mice hung passively and remained still were they deemed immobile [39].

### 2.10. Sedative Activity

#### 2.10.1. HCT

The approach was used as Takagi et al. (1971) had described. A cage measuring 30 × 20 × 14 cm with a divider fastened in the center. At a height of 7.5 cm, a 3‐cm‐diameter opening was created in the middle of the cage. Mice were put on one side of the cage and given either extract, standard, or control treatment. After the standard diazepam (po) and test SAL‐ME (po) medications were administered, the number of times a mouse passed through the hole from one chamber to the next was then counted for 3 min at 0, 30, 60, 90, and 120 min [40].

#### 2.10.2. OFT

This test was carried out as described by Gupta et al. A half‐square‐meter wooden field with several squares alternately painted in black and white made up the open field equipment. It was situated in a poorly lit chamber with a wall that was 50 cm high. Mice were placed in the center of the open field and given either vehicle, extract, or diazepam. The animals’ visits to the squares were then counted for three minutes at 30, 60, 90, and 120 min following the treatments [41, 42].

### 2.11. Antipyretic Activity

#### 2.11.1. Brewer’s Yeast‐Induced Pyrexia in Mice

A 20% (W/V) brewer’s yeast suspension (10 mL/kg) was subcutaneously injected into the animal’s dorsum area to cause pyrexia. There were four groups of five animals apiece. After that, the animals were given unrestricted access to water and fasted for about 24 h. To ascertain the pyretic reaction to yeast, control temperatures were recorded 18 h following the injection. Mice that displayed a temperature increase of at least 0.6°C were selected for the investigation. Following an 18‐h yeast injection, the extract SAL‐ME (200 and 400 mg/kg) and the conventional medication (paracetamol, 100 mg/kg) were administered orally. The mice’s initial rectal temperatures were observed at 0 h. Temperature readings were taken one to 4 h following the medication administration [43, 44].

### 2.12. Molecular Docking Study

#### 2.12.1. Assessment of the Ligands’ Absorption, Distribution, Metabolism, Excretion, and Toxicity (ADMET) Characteristics

The identification of candidate compounds as potential therapeutic agents is primarily guided by their physicochemical and molecular properties, alongside key pharmacokinetic parameters, including ADMET. The pharmacokinetic profiles of the selected ligands were evaluated using the pKCSM online platform (http://biosig.unimelb.edu.au/pkcsm/) as described by Pires, Blundell, and Ascher (2015). Additionally, Lipinski’s Rule of Five was employed via the SwissADME web server to assess the drug‐likeness and oral bioavailability of the identified compounds [45].

#### 2.12.2. Protein Preparation

The crystallographic data for the target proteins were accessed via the RCSB Protein Data Bank. Proteins used in the docking study included monoamine oxidase (PDB: 2Z5X) [46], serotonin transporter (PDB: 5I6X), a GABA_A_ receptor isoform (α1β2γ2, PDB: 6X3T), and microsomal prostaglandin E (PGE) synthase‐1 (PDB: 4YK5). These structural datasets were originally reported by Kurumbail and colleagues in their earlier studies [47]. The target protein underwent essential preprocessing steps. Water molecules, heteroatoms, and cofactors were removed during structural preparation using Swiss‐PdbViewer (v4.1) alongside BIOVIA Discovery Studio 4.5 Client. Following the addition of hydrogen atoms, energy minimization of the protein structure was carried out through the MMFF94 force field within the PyRx virtual screening environment [26, 48]. For molecular docking studies, the target protein structure was saved in .pdb (Protein Data Bank) format to ensure compatibility with docking software.

#### 2.12.3. Selection of Ligands

A total of 9 compounds previously identified through GC–MS/MS analysis were taken and presented in Supporting Figure 1 and were selected for further investigation [49]. The 3D structures of these compounds were obtained in SDF format from the PubChem database. For compounds that lacked available 3D conformers, their 2D SDF representations were converted to 3D using Open Babel software [50].

#### 2.12.4. Molecular Docking and Postdocking Analysis

Docking procedures were conducted utilizing AutoDock Version 4.2 in conjunction with PyRx 0.8 (available at http://pyrx.scripps.edu) to perform virtual screening and evaluate ligand–protein binding interactions [51, 52]. AutoGrid was employed to generate the grid box for docking. The resulting docking conformations were analyzed using PyMOL, which enabled visualization and evaluation of key molecular interactions involved in ligand binding. Additional insights into receptor–ligand interactions were also obtained through PyMOL.

### 2.13. Prediction of Activity Spectra for Substances (PASS)

At http://www.pharmaexpert.ru/passonline/predict.php, the PASS online tools were employed to examine the PASS prediction and ascertain the possible biological impacts of the chemicals that were chosen. Probability of inactivity (Pi) and probability of activity (Pa) had values between 0.000 and 1.000 [53]. Biological activity is predicted when the Pa exceeds the Pi. Low pharmaceutical activity is indicated by Pa < 0.5, moderate therapeutic potential is suggested by Pa < 0.5, and high medicinal activity is indicated by Pa > 0.7 [54, 55].

### 2.14. Statistical Analysis

The experimental data were expressed as mean ± standard error of the mean (SEM). Statistical analysis was conducted using SPSS software (Version 16.0, IBM Corp., New York, USA). Group comparisons were performed through one‐way ANOVA, followed by Dunnett’s post hoc test to assess statistical significance. Graphs and additional visualizations were created using “Microsoft Excel 2021 and GraphPad Prism 8.0.1.” The chemical structures of the identified phytocompounds were illustrated using “ChemDraw Ultra 12.0.2.” Statistical significance was interpreted through the following criteria: ^∗^ *p* < 0.05, ^∗∗^ *p* < 0.01, and ^∗∗∗^ *p* < 0.001, with significant outcomes denoting a difference from the control group.

## 3. Results

### 3.1. Acute Oral Toxicity

At the test doses, no harm was detected. Additionally, throughout this toxicity test, none of the mice perished. Variations in the animals’ daily food and water intake were managed. Therefore, it was shown that the extract SAL‐ME’s LD_50_ was higher than 4000 mg/kg. This demonstrates that the extract remained stable up to 4000 mg/kg after a single bodyweight dosing. For the pharmacological action, 200 and 400 mg/kg BW, po doses were chosen based on the findings of the acute oral toxicity test and the safety.

### 3.2. Anxiolytic Activity

#### 3.2.1. EPM Test

**FIGURE 1:**
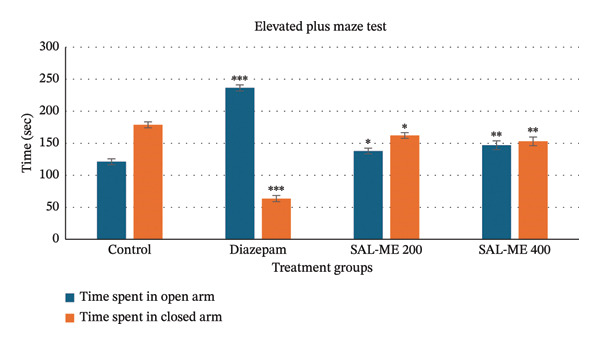
Determination of anxiolytic activity of SAL‐ME through EPM.

SAL‐ME exhibited mild, dose‐dependent anxiolytic activity in the EPM test. At 400 mg/kg, treated mice spent significantly more time in the open arms (147 ± 6.78 s; *p* < 0.01) and showed a moderate increase in open arm entries (*p* < 0.01) relative to the control, though this effect was markedly less pronounced than that of diazepam (236.4 ± 4.77 s; *p* < 0.001). The 200 mg/kg dose produced weaker anxiolytic responses, underscoring the dose‐dependent nature of the effect (Table 1, Figure 1).

**TABLE 1:** Effect of SAL‐ME extract on elevated plus maze test.

Treatment (mg/kg)	Time spent in open arms (s)	Entries in open arms	Time spent in close arms (s)	Entries in close arms
Control	121.2 ± 4.57	6.4 ± 1.03	178.8 ± 4.57	11.6 ± 1.21
Diazepam	236.4 ± 4.77^∗∗∗^	13 ± 1.14^∗∗∗^	63.6 ± 4.77^∗∗∗^	6.2 ± 0.58^∗∗^
SAL‐ME 200	137.8 ± 4.47^∗^	10.4 ± 1.12	162.2 ± 4.47^∗^	8.6 ± 0.81
SAL‐ME 400	147 ± 6.78^∗∗^	11.2 ± 0.37^∗∗^	153 ± 6.78^∗∗^	7 ± 0.71^∗^

*Note:* All values are shown as Mean ± SEM, and statistical analysis is done using one‐way analysis of variance (ANOVA). Subsequently, *n* = 5 is used for Dunnett′s multiple‐comparison test, with ^∗^ *p* < 0.05, ^∗∗^ *p* < 0.01, and ^∗∗∗^ *p* < 0.001 compared with the control group.

#### 3.2.2. HBT

**FIGURE 2:**
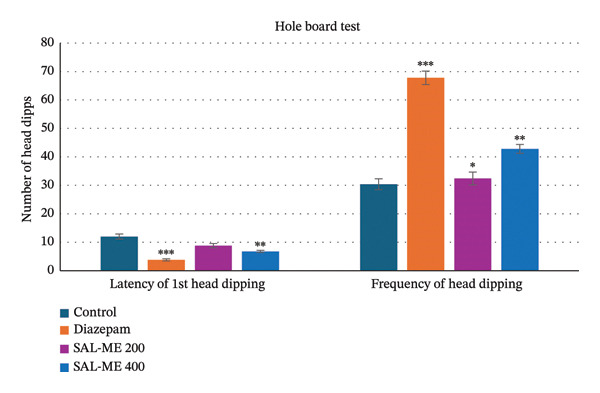
Determination of anxiolytic activity of SAL‐ME through HBT.

In this test, head‐dipping behavior increased with higher dosages. At 400 mg/kg, SAL‐ME showed moderate (*p* < 0.01) head dipping behavior compared to the standard (*p* < 0.001). Moderately increased head dipping behavior (Figure 2).

### 3.3. Antidepressant Activity

#### 3.3.1. FST

**FIGURE 3:**
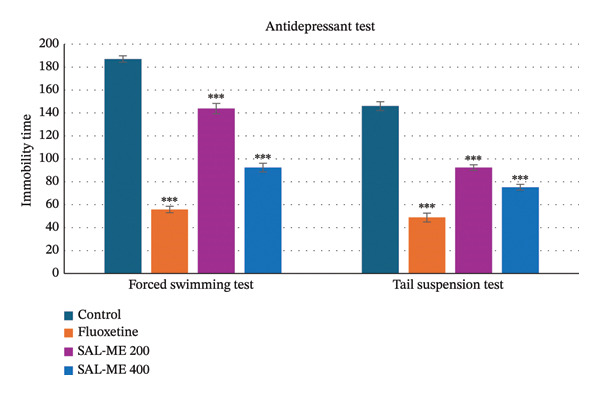
Determination of antidepressant activity of SAL‐ME through forced swimming and tail suspension tests.

In this test, immobility time decreased significantly with increasing doses. The extract markedly reduced immobility time at 200 and 400 mg/kg (143.8 ± 4.47 and 92.4 ± 3.74 s, respectively; *p* < 0.001). Similarly, the standard drug fluoxetine significantly decreased immobility time (55.8 ± 2.82, *p* < 0.001) (Figure 3).

#### 3.3.2. TST

The extract also demonstrated strong antidepressant effects in this test. The immobility time of the mice was significantly shortened by the standard drug fluoxetine (48.8 ± 3.87 s, *p* < 0.001). SAL‐ME at both doses significantly decreased immobility time compared to the control. The results are presented in Figure 3.

### 3.4. Sedative Activity

#### 3.4.1. HCT

As shown in Table 2, the frequency of mice moving between chambers decreased over 2 h during the hole‐cross test. The hole‐cross method revealed that SAL‐ME at 200 and 400 mg/kg BW reduced locomotor activity in experimental animals. At 400 mg/kg, the extract significantly decreased movement frequency from 30 to 120 min (*p* < 0.01). This finding suggests a sedative effect, as the extract also reduced exploratory behavior, indicating its potential to decrease overall activity.

**TABLE 2:** Determination of sedative activity of SAL‐ME through the hole cross test.

Treatment dose (mg/kg)	Number of squares crossed				
	0 min	30 min	60 min	90 min	120 min
Control	19.2 ± 0.97	17.6 ± 0.98	14.6 ± 0.93	13.6 ± 1.08	10.2 ± 0.8
Diazepam	17.6 ± 1.17	13 ± 0.71^∗^	9.2 ± 0.49^∗∗∗^	7 ± 0.63^∗∗^	4.6 ± 0.51^∗∗^
SAL‐ME 200	19 ± 1.05	16.6 ± 1.33	14.8 ± 0.86	11.4 ± 0.6	8 ± 0.77
SAL‐ME 400	20 ± 1.22	16.2 ± 0.66	12.4 ± 0.93^∗∗^	8.6 ± 0.68^∗∗^	5.6 ± 0.51^∗∗^

*Note:* All values are shown as Mean ± SEM, and statistical analysis is done using one‐way analysis of variance (ANOVA). Subsequently, *n* = 5 is used for Dunnett′s multiple‐comparison test, with ^∗^ *p* < 0.05, ^∗∗^ *p* < 0.01, and ^∗∗∗^ *p* < 0.001 compared with the control group.

#### 3.4.2. OFT

**FIGURE 4:**
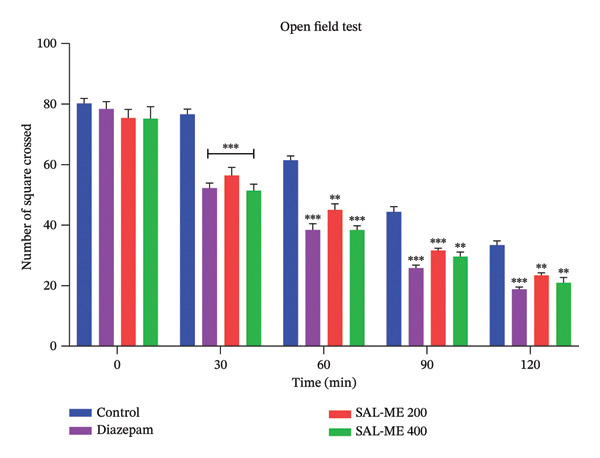
Determination of sedative activity of SAL‐ME through OFT.

Findings from the OFT demonstrate that the extract has a sedative effect. At a dosage of 400 mg/kg, a marked reduction in movement count was observed at 30 min (51.4 ± 2.11) and 60 min (38.4 ± 1.36), with statistical significance (*p* < 0.001). These effects were closely aligned with those produced by the standard drug, which showed movement counts of (52.2 ± 1.69) and (38.4 ± 2.09) at the corresponding time points. The control group showed higher activity (76.6 ± 1.72 and 61.4 ± 1.44 movements). Similarly, the 200 mg/kg dose also reduced movements at both time points (*p* < 0.001). The observed decline in locomotor activity and rearing behavior indicates the extract’s potential sedative properties (Figure 4).

### 3.5. Antipyretic Activity

#### 3.5.1. Brewer’s Yeast‐Induced Pyrexia

Mice injected subcutaneously with yeast suspension exhibited elevated body temperatures after 24 h. However, SAL‐ME at 400 mg/kg significantly (*p* < 0.01) reduced body temperature to 98.12 ± 0.44 °F after 180 min (Table 3). Paracetamol, the standard drug, effectively reduced fever across all time frames. In comparison to the fever control, SAL‐ME demonstrated antipyretic effects that persisted for 2 h posttreatment.

**TABLE 3:** Effect of SAL‐ME on yeast‐induced pyrexia in mice.

Treatment dose (mg/kg)	Normal rectal temperature (°F)	Temperature after pyrexia (°F)	Rectal temperature (°F) after the drug administration		
			60 min	120 min	180 min
Control	98.21 ± 0.66	102.43 ± 0.54	101.21 ± 0.44	100 ± 1.16	99.2 ± 0.54
Paracetamol	97.8 ± 0.88	103.32 ± 1.44	99.23 ± 0.66^∗∗^	98.56 ± 0.88^∗∗^	97.32 ± 0.44^∗∗^
SAL‐ME 200	98.1 ± 1.24	102.13 ± 0.66	102.11 ± 1.66	101.21 ± 1.24^∗^	100 ± 0.24
SAL‐ME 400	97.55 ± 0.44^∗^	104.1 ± 0.54^∗∗^	102.21 ± 0.66	100.45 ± 1.16	98.12 ± 0.44^∗∗^

*Note:* All values are shown as Mean ± SEM, and statistical analysis is done using one‐way analysis of variance (ANOVA). Subsequently, *n* = 5 is used for Dunnett’s multiple comparison test, with ^∗^ *p* < 0.05, ^∗∗^ *p* < 0.01, and ^∗∗∗^ *p* < 0.001 compared with the control group.

### 3.6. In Silico Study

#### 3.6.1. Determination of ADMET Properties and Pharmacokinetic Parameters

The evaluation revealed that all selected molecules complied with Lipinski’s Rule of Five, indicating their potential suitability for oral administration. Additionally, the admetSAR online database (http://lmmd.ecust.edu.cn/admetsar2/) was used to predict the toxicological profiles of the 24 substances. The findings of the analysis indicated that the compounds were noncarcinogenic and non‐Ames toxic, supporting their safety for further pharmacological investigation (Table 4).

**TABLE 4:** ADMET and pharmacokinetic analysis of SAL‐ME compounds.

Compound name	Lipinski rules					Lipinski’s Violation ≤ 1	Veber’s rules		Toxicity and pharmacokinetic parameters				
	MW (g/mol) < 500	HBA < 10	HBD < 5	Log *p* ≤ 5	Bioavailability score		*n* RB ≤ 10	TPSA ≤ 140 (Å^2^)	Ames toxicity	Carcinogens	Acute oral toxicity	Human intestinal absorption	Blood–brain barrier (BBB)
Butanoic acid	88.11	2	1	1.1	0.85	0	2	37.30	NAT	NC	III	0.9938	0.9572
Glycerol	92.09	3	3	0.45	0.55	0	2	60.69	NAT	NC	IV	0.9239	0.6136
Glyceric acid	106.08	4	3	−0.54	0.56	0	2	77.76	NAT	NC	IV	0.741	0.5927
Butanedioic acid	118.09	4	2	0.32	0.85	0	3	74.60	NAT	NC	III	0.668	0.869
D‐Pinitol	194.18	6	5	−0.24	0.55	0	1	110.38	NAT	NC	III	0.8097	0.6587
Myo‐inositol	180.16	6	6	0.31	0.55	1	0	121.38	NAT	NC	III	0.8654	0.5194
Hexadecanoic acid	256.42	2	1	3.85	0.85	1	14	37.30	NAT	NC	IV	0.9888	0.9488
Octadecanoic acid	284.48	2	1	4.3	0.85	1	16	37.30	NAT	NC	IV	0.9888	0.9488
α‐D‐Glucopyranoside	194.18	6	4	0.8	0.55	0	2	99.38	NAT	NC	III	0.5308	0.508

#### 3.6.2. Molecular Docking Study

**FIGURE 5:**
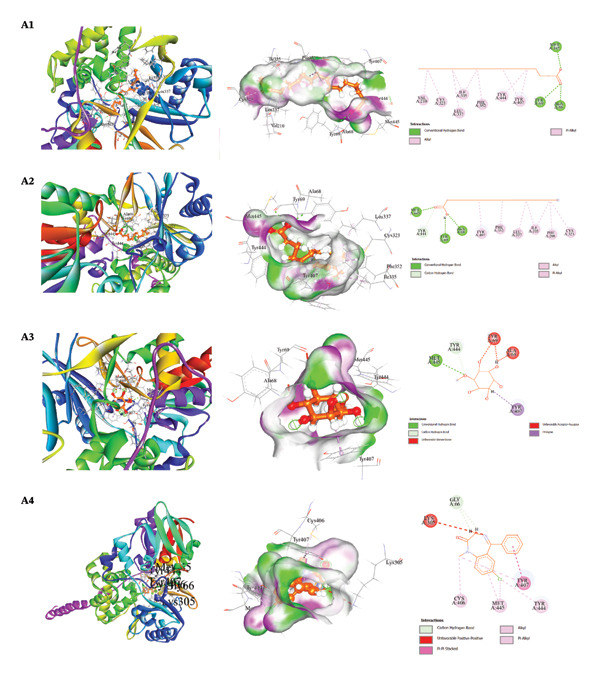
Analysis of molecular docking interactions between selected compounds and the human monoamine oxidase enzyme (PDB ID: 2Z5X): **A1**. octadecanoic acid, **A2**. hexadecanoic acid, **A3**. D‐pinitol, and **A4**. diazepam (standard).

**FIGURE 6:**
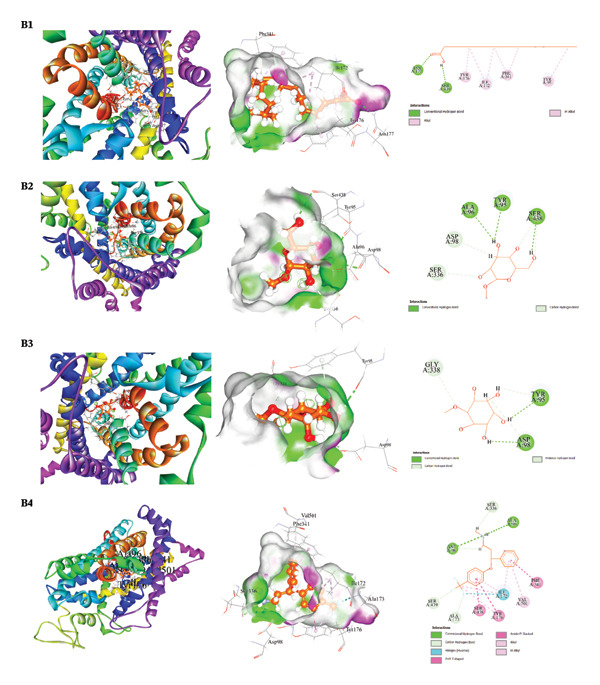
Analysis of molecular docking interactions between the human serotonin transporter (PDB: 5I6X): **B1**. octadecanoic acid, **B2**. α‐D‐glucopyranoside, **B3**. D‐pinitol, and **B4**. fluoxetine (standard).

**FIGURE 7:**
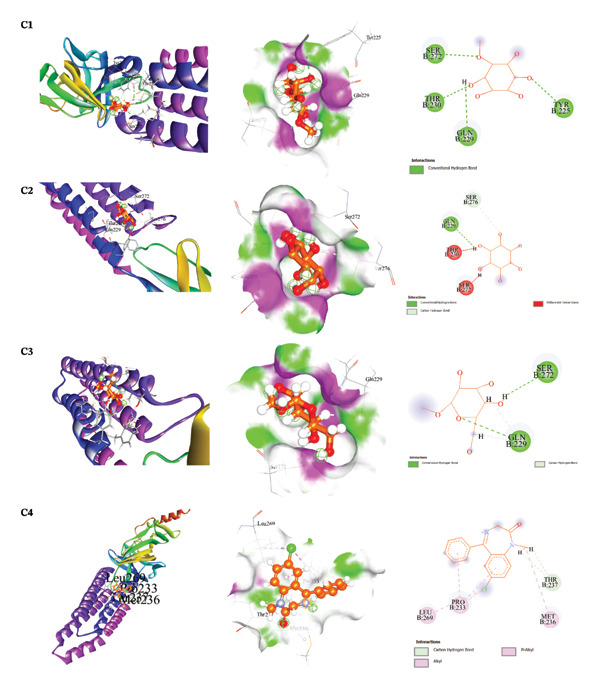
Analysis of molecular docking interactions between the human GABA_A_ receptor isoform (α1β2γ2, PDB: 6X3T): **C1**. D‐pinitol, **C2**. myo‐inositol, **C3**. α‐D‐glucopyranoside, and **C4**. diazepam (standard).

**FIGURE 8:**
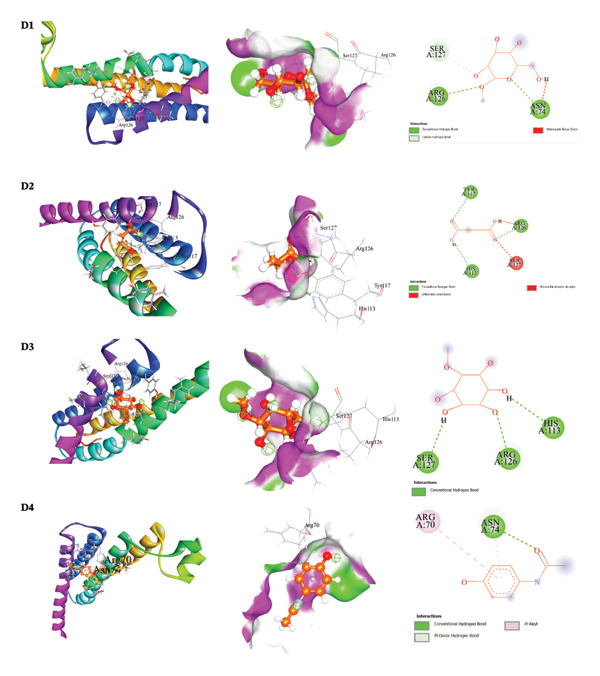
Analysis of molecular docking interactions between the microsomal PGE synthase 1 (PDB: **4YK5**): **D1**. α‐D‐glucopyranoside, **D2**. butanedioic acid, **D3**. D‐pinitol, and **D4**. Paracetamol (standard).

The docking results and interaction analyses for the top three SAL‐ME compounds associated with each biological activity, along with the respective reference drugs targeting the same proteins, are illustrated in Figures 5, 6, 7, 8 and detailed in​ Table 5. An overall summary of docking scores for all activities is presented in Table 6.

**TABLE 5:** SAL‐ME’s selected phytochemicals in silico binding affinity and nonbonding interaction for anxiolytic (2Z5X), antidepressant (5I6X), sedative (6X3T), and antipyretic (4YK5) properties, respectively.

Section number	Receptor	Compound	Binding affinity (kcal/mol)	Bond type	Amino acids
1	**2Z5X**	Octadecanoic acid	−7.4	CHB	ALA68, TYR69, MET445
				A	ILE335, ILE335, LEU337, VAL210, CYS323
				P–A	PHE352, TYR407, TYR444
		Hexadecanoic acid	−7.3	CHB	ALA68, MET445, TYR69
				Ca–HB	TYR444
				A	ILE335, LEU337, CYS323
				P–A	ILE335, PHE208, PHE352, TYR407
		D‐Pinitol	−6	Pi–Sigma	TYR407
				CHB	MET445
				Ca–HB	TYR444
		Diazepam (standard)	−8.8	Ca–HB	GLY66
				Pi–Pi stacked	TYR407
				A	CYS406, MET445
				P–A	TYR407, TYR444, MET445

2	**5I6X**	Octadecanoic acid	−6.5	CHB	ASN177, SER439
				A	ILE172
				P–A	TYR95, TYR176, PHE341
		α‐D‐Glucopyranoside	−6	CHB	SER438, TYR95, ALA96
				Ca–HB	SER336, SER438, ASP98
		D‐Pinitol	−5.7	CHB	ASP98, TYR95
				Ca–HB	GLY338, ASP98
				Pi–donor hydrogen bond	TYR95
		Fluoxetine (standard)	−9.2	CHB	ALA96, ASP98
				Ca–HB; halogen (fluorine)	ALA173, SER439
				Ca–HB	ASP98, SER336
				Halogen (fluorine)	ILE172, SER439
				Pi–Pi T‐shaped	TYR176, PHE341
				Amide–Pi Stacked	SER438, SER439
				A	ILE172
				P–A	TYR176, ILE172, VAL501

3	**6X3T**	D‐Pinitol	−4.6	CHB	TYR225, THR230, SER272, GLN229
		Myo‐inositol	−4.5	CHB	GLN229
				Ca–HB	SER276
		α‐D‐Glucopyranoside	−4.3	CHB	GLN229, SER272
				Ca–HB	GLN229
		Diazepam (standard)	−4.9	CHB	THR237
				A	MET236, PRO233, LEU269
				P–A	PRO233

4	**4YK5**	α‐D‐Glucopyranoside	−4.4	CHB	ASN74, ARG126
				Ca–HB	SER127
		Butanedioic acid	−4.4	CHB	TYR117, ARG126, HIS113
		D‐Pinitol	−4.3	CHB	ARG126, SER127, HIS113
		Paracetamol (standard)	−4	CHB	ASN74
				Pi–donor hydrogen bond	ASN74
				P–A	ARG70

*Note:* Ca–HB = carbon–hydrogen bond, A = alkyl, P–A = Pi–alkyl.

Abbreviation: CHB, conventional hydrogen bond.

**TABLE 6:** Binding scores of the reported phytochemicals of SAL‐ME.

Compounds	PubChem CID	Docking score (kcal/mol)			
		Anxiolytic (2Z5X)	Antidepressant (5I6X)	Sedative (6X3T)	Antipyretic (4YK5)
Butanoic acid	264	−4.1	−4.3	−3	−3.4
Glycerol	753	−3.6	−3.8	−2.9	−3.2
Glyceric acid	752	−4.5	−4.1	−3.4	−3.7
Butanedioic acid	1110	−4.6	−4.6	−3.7	−4.4
D‐Pinitol	164,619	−6	−5.7	−4.6	−4.3
Myo‐inositol	892	−5.7	−5.5	−4.5	−4.3
Hexadecanoic acid	985	−7.3	−6.1	−3.2	−3.8
Octadecanoic acid	5281	−7.4	−6.5	−2.6	−3.7
α‐D‐Glucopyranoside	64,947	−5.4	−6	−4.3	−4.4
Standard (diazepam, fluoxetine, diazepam, and paracetamol)		−8.8	−9.2	−4.9	−4

##### 3.6.2.1. Molecular Docking for Anxiolytic Activity

The anxiolytic potential of selected bioactive constituents of SAL‐ME was evaluated through molecular docking against human monoamine oxidase‐A (MAO‐A; PDB ID: 2Z5X). All tested compounds demonstrated appreciable binding affinity toward the target receptor. Among them, octadecanoic acid (−7.4 kcal/mol) and hexadecanoic acid (−7.3 kcal/mol) exhibited the strongest binding energies, followed by D‐pinitol (−6.0 kcal/mol). However, these binding scores were comparatively lower than that of the standard drug diazepam (−8.8 kcal/mol). Interaction analysis revealed that the fatty acids predominantly engaged in hydrophobic and π–alkyl interactions with key amino acid residues within the MAO‐A active site, suggesting favorable ligand–receptor stabilization. The observed interaction patterns partially overlapped with those of diazepam, indicating their potential contribution to anxiolytic activity (Table 5, Section 1, Figure 5).

##### 3.6.2.2. Molecular Docking for Antidepressant Activity

Docking analysis against the human serotonin transporter (PDB ID: 5I6X) demonstrated that all selected phytochemicals possessed varying degrees of antidepressant potential. Octadecanoic acid showed the highest binding affinity among the tested compounds (−6.5 kcal/mol), followed by α‐D‐glucopyranoside (−6.0 kcal/mol) and D‐pinitol (−5.7 kcal/mol). Nevertheless, these values were lower than that of the reference drug fluoxetine (−9.2 kcal/mol). Detailed interaction profiling indicated that the ligands formed a combination of hydrophobic, hydrogen‐bond, and halogen interactions with critical residues in the transporter binding pocket, supporting their moderate binding stability and possible antidepressant relevance (Table 5, Section 2, Figure 6).

##### 3.6.2.3. Molecular Docking for Sedative Activity

The sedative potential of SAL‐ME constituents was evaluated by docking against the human GABAA_AA receptor isoform (α1β2γ2; PDB ID: 6X3T). Among the tested compounds, D‐pinitol exhibited the strongest binding affinity (−4.6 kcal/mol), followed by myo‐inositol (−4.5 kcal/mol) and α‐D‐glucopyranoside (−4.3 kcal/mol). These values were comparable, though slightly lower, than the binding energy of the standard drug diazepam (−4.9 kcal/mol). Interaction analysis revealed that these compounds mainly formed hydrogen bonds and hydrophobic interactions with amino acid residues located within the receptor’s active site, suggesting a plausible role in modulating sedative effects (Table 5, Section 3, Figure 7).

##### 3.6.2.4. Molecular Docking for Antipyretic Activity

For antipyretic activity, docking studies were conducted against microsomal PGE synthase‐1 (PDB ID: 4YK5). Butanedioic acid and α‐D‐glucopyranoside both showed the highest binding affinity (−4.4 kcal/mol), followed closely by D‐pinitol (−4.3 kcal/mol). These binding energies were comparable to those of the standard antipyretic drug paracetamol (−4.0 kcal/mol). Further analysis indicated that these compounds interacted with key amino acid residues through hydrogen bonding and hydrophobic interactions at short intermolecular distances, supporting their affinity for the enzyme’s active site and their potential contribution to antipyretic activity (Table 5, Section 4, Figure 8).

### 3.7. PASS Prediction

Twenty‐four compounds of SAL‐ME were screened through PASS prediction analysis for anxiolytic, antidepressant, sedative, and antipyretic activities. The results are depicted in Table 7.

**TABLE 7:** PASS prediction of biologically active compounds of SAL‐ME.

Compound name	Biological activity							
	Anxiolytic		Antidepressant		Sedative		Antipyretic	
	Pa	Pi	Pa	Pi	Pa	Pi	Pa	Pi
Butanoic acid	0.292	0.067	0.123	0.111	0.221	0.058	**0.505**	0.013
Glycerol	0.076	0.038	**0.210**	0.055	**0.392**	0.004	0.412	0.024
Glyceric acid	0.187	0.164	—	—	0.380	0.004	0.313	0.041
Butanedioic acid	0.305	0.059	0.179	0.071	0.219	0.059	0.473	0.017
D‐Pinitol	0.244	0.102	—	—	0.128	0.116	0.239	0.081
Myo‐inositol	**0.330**	0.045	0.182	0.069	0.183	0.089	0.274	0.056
α‐D‐Glucopyranoside	—	—	—	—	0.186	0.075	0.183	0.132
Hexadecanoic acid	0.261	0.088	—	—	0.204	0.070	0.497	0.013
Octadecanoic acid	0.261	0.088	—	—	0.204	0.070	0.497	0.013

*Note:* Bold values indicate the highest predicted activity for each category.

## 4. Discussion

Natural products have attracted increasing global attention due to their structural diversity, broad spectrum of bioactive constituents, and generally favorable safety profiles with low toxicity [56]. Since ancient times, plants have played a pivotal role in traditional medicine, serving as a primary source of therapeutic agents for the prevention and treatment of diverse human ailments [57]. Many ethnomedicinal plants are known to enhance neurobehavioral states and serve as alternatives to modern medicine. Our study aimed to investigate the CNS and antipyretic effects of SAL‐ME. The results demonstrated that SAL‐ME exhibits significant antidepressant and sedative effects, along with moderate anxiolytic activity on the central nervous system. Oral SAL‐ME dosing did not result in behavioral problems, allergic responses (skin rash, itching), or mortality throughout the 72‐h monitoring period in acute toxicity studies. SAL‐ME is therefore clearly nontoxic at the studied dosages.

The EPM test is a widely validated animal model for evaluating the anxiolytic activity of substances [58]. In this study, extract‐treated animals exhibited dose‐dependent mild anxiolytic effects by spending more time in the open arms and increasing the number of entries into the open arms compared to the control group. Previous research highlights that the time spent in the light region is a more reliable parameter for assessing anxiolytic activity than the number of entries [59]. Further supporting the anxiolytic qualities of *S. asper*, SAL‐ME at 400 mg/kg moderately (*p* < 0.01) increased the amount of time spent in the light region compared to the control. Anxiolytic drugs typically act by enhancing GABA responses through the opening of GABA‐activated chloride ion channels. Based on these findings, it may be hypothesized that *S. asper* exerts effects similar to benzodiazepines [60]. A straightforward technique for evaluating how animals react emotionally and anxiously to unfamiliar situations is the hole‐board test [61]. A decrease in head‐dipping behavior is indicative of CNS depressive activity in animal models [62]. In the current study, *S. asper* demonstrated dose‐dependent CNS depressive effects, as evidenced by a significant (*p* < 0.01) reduction in head dips compared to the control group.

Depression and anxiety are closely linked conditions that often coexist, and addressing both enhances therapeutic effectiveness. Serotonergic antidepressants, or SSRIs, are the first‐line treatment for depression and have notable anxiolytic benefits as well [63]. Recent studies have explored the impact of medications influencing serotonin (5‐HT) transmission on depressive behavior [64]. Similarly, *S. asper* demonstrated both anxiolytic and antidepressant activities in this study. In the FST and TST, immobility or hopelessness in animals reflects behaviors analogous to human depression [65]. In the FST, prolonged immobility indicates CNS depression‐like effects, while reduced immobility suggests antidepressant activity. The TST serves as a rapid and precise method to assess psychotropic effects, measuring the energy expended by mice as they struggle to escape suspension [66]. In this study, the extract SAL‐ME exhibited dose‐dependent reduction of immobility time compared to the control. At both 200 mg/kg and 400 mg/kg doses, SAL‐ME significantly reduced immobility times in the FST (143.8 ± 4.47 and 92.4 ± 3.74 s, respectively) and TST (92.4 ± 2.4 and 75.2 ± 2.5 s, respectively) compared to the control group (*p* < 0.001). The higher dose caused a shorter immobility period than the lower dose (200 mg/kg), according to statistical analysis, demonstrating its better antidepressant effectiveness. These findings suggest that the 400 mg/kg dose of the SAL‐ME crude extract is more effective as an antidepressant.

At doses of 200 and 400 mg/kg BW, the extract SAL‐ME significantly reduced rearing behavior in the closed arms of the EPM. Similarly, in the OFT, rearing decreased at these doses, comparable to the positive control. According to Rodgers et al. [31], this reduction in rearing indicates a decline in locomotor activity, potentially attributable to the sedative properties of the plant. The sedative effects of *S. asper* may be linked to the presence of certain components in the decoction that activate benzodiazepine and/or GABA receptors within the GABA receptor complex [67, 68].

We investigated the sedative properties of SAL‐ME using the hole‐cross test in animals. The findings demonstrated that the extract dose‐dependently reduced the number of movements through the hole. A sign of the excitability of the central nervous system is locomotor activity. Substances that significantly reduce mobility are interpreted as sedatives in the HCT because they indicate a decrease in curiosity and exploratory behavior in a novel setting [69]. The marked reduction in spontaneous motor activity observed in this study suggests the sedative activity of the plant extract [70]. In the present study, the locomotor activity‐lowering effect became evident during the 3rd observation period (60 min) and persisted until the 5th observation period (120 min) in mice. Notably, SAL‐ME at 400 mg/kg produced highly significant effects (*p* < 0.01) compared to SAL‐ME at 200 mg/kg, indicating a dose‐dependent sedative action.

In a previous study, the ethanolic leaf extract of *S. asper* demonstrated neuroprotective potential by significantly attenuating glutamate‐induced toxicity in HT22 hippocampal neuronal cells in a dose‐dependent manner [71]. Furthermore, its basic and neutral fractions exhibited dose‐dependent cholinesterase inhibitory activity, suggesting therapeutic relevance in Alzheimer’s disease management [72]. The extract also shows promise in mitigating other neurodegenerative conditions, including Parkinson’s disease [73]. Notably, a dose‐dependent reduction in epileptic seizures was observed—potentially attributed to betulin, identified in the bark extract, which may cross the blood–brain barrier and exert anticonvulsant effects via competitive binding to the GABA_A_ receptor [74]. Collectively, these findings highlight *S. asper* as a multitarget candidate for neurological disorder interventions.

The study provided significant insights into the antipyretic activity of SAL‐ME through biological and computational evaluations of selected compounds from *S. asper*. Similar to paracetamol, antipyretic drugs like SAL‐ME may work by inhibiting cyclooxygenase enzymes, which would inhibit the creation of prostaglandins [75]. This inhibition prevents the production of pyrexia‐inducing mediators, contributing to the observed antipyretic effect [76]. Since both the paracetamol and SAL‐ME dose‐dependently decreased the rectal temperature of yeast‐induced febrile mice, it is conceivable that the antipyretic action of SAL‐ME is caused by the presence of bioactive phytochemicals that block prostaglandin synthesis, similar to other analgesic medications. Additionally, nonsteroidal anti‐inflammatory drugs (NSAIDs) are known to exhibit antipyretic effects by inhibiting prostaglandin synthesis within the hypothalamus [77]. Thus, it can be inferred that SAL‐ME may exert its antipyretic effects through a similar mechanism involving the suppression of prostaglandin production. In a prior study, the hydroalcoholic extract of *S. asper* leaves demonstrated significant antipyretic activity in the brewer’s yeast‐induced pyrexia model [78]—a finding that aligns well with the antipyretic effect observed for our SAL‐ME, supporting the consistency and extract‐type‐independent bioactivity of this plant.

Clarifying ligand‐target interactions, describing how tiny molecules behave within target protein binding sites, and comprehending the mechanisms driving diverse pharmacological reactions all depend on molecular docking [79]. In the present study, docking analysis was employed to rationalize the bioactivities of SAL‐ME against four key protein targets associated with anxiolytic, antidepressant, sedative, and antipyretic effects. Several phytocompounds, notably octadecanoic acid, hexadecanoic acid, D‐pinitol, α‐D‐glucopyranoside, myo‐inositol, and butanedioic acid, demonstrated appreciable binding affinities with their respective receptors. Strong interactions of octadecanoic and hexadecanoic acids with MAO‐A suggest a possible role in anxiolytic activity, whereas D‐pinitol and myo‐inositol showed favorable binding toward the GABA_A_ receptor, supporting sedative potential. Moreover, butanedioic acid and α‐D‐glucopyranoside exhibited binding affinities comparable to paracetamol against microsomal PGE synthase‐1. Collectively, these findings indicate that the pharmacological effects of SAL‐ME are likely mediated through the concerted and synergistic actions of multiple bioactive constituents, providing molecular‐level support for the experimental observations.

To find theoretically active principles with appealing ADMET profiles that have been isolated before but have not yet been tested for activity against specific therapeutic targets, virtual screening is crucial for natural product researchers [80]. Compared to random screening, this prediction process might be a better choice for lead search. Based on the ADMET test results, it is evident that all the compounds met Lipinski’s rule of five, which calls for a drug to have better pharmacokinetics and less toxicity.

## 5. Limitations and Future Directions

While the present findings are promising, the crude nature of the extract prevents identification of which compounds are primarily responsible for the observed effects. Further studies should involve fractionation, isolation, and structural elucidation of active constituents, receptor‐binding assays, chronic toxicity studies, and mechanistic evaluations using antagonists or knockout models. Clinical translation will require thorough pharmacological and safety profiling.

## 6. Conclusions

The SAL‐ME demonstrated significant anxiolytic, antidepressant, sedative, and antipyretic properties, likely mediated through GABAergic modulation, serotonergic pathways, and prostaglandin inhibition. Both in vivo behavioral assays and in silico docking analyses highlight octadecanoic acid, hexadecanoic acid, D‐pinitol, α‐D‐glucopyranoside, myo‐inositol, and butanedioic acid as promising bioactive compounds with favorable drug‐likeness and safety profiles.

These findings provide scientific validation for the ethnomedicinal uses of *S. asper* and underscore its potential as a source of multitarget phytopharmaceuticals for CNS disorders and fever. Future work focusing on isolation, characterization, and detailed mechanistic studies of active phytochemicals is warranted to advance the development of *S. asper*–based therapeutics.

## Author Contributions

Md. Hossain Rasel: conceptualization; data curation; formal analysis; investigation; methodology; software; writing–original draft (lead); writing–review and editing. Md. Jahirul Islam Mamun: data analysis; writing; investigation; software; writing–review and editing. Salma Sultana Sumi: data analysis; writing; software. Md. Nahid Hasan: data analysis; writing; software. Minhajur Rahman: data interpretation; writing–review and editing. S. M. Naim Uddin: supervision, data analysis; data interpretation; writing–review and editing.

## Funding

No funding was received for this manuscript.

## Conflicts of Interest

The authors declare no conflicts of interest.

## Supporting Information

The Supporting Information for this article includes one additional figure. Supporting Figure 1 presents the phytochemical structures of the identified SAL‐ME compounds. These structural data complement the GC–MS findings and support the subsequent computational and pharmacological analyses described in the main manuscript.

## Supporting information


**Supporting Information** Additional supporting information can be found online in the Supporting Information section.

## Data Availability

The data supporting the study’s findings will be made available from the corresponding author upon reasonable request.
