# Supplementary material for: Neuropharmacological and Antipyretic Potentials of Streblus asper Leaves: Integrated In Vivo and In Silico Approaches
Source: ScientificWorldJournal. 2026 Apr 13;2026:7661411. doi: 10.1155/tswj/7661411 (PMC13071759; doi:10.1155/tswj/7661411)
Supplement: Supplementary file 1 — Supporting Information Additional supporting information can be found online in the Supporting Information section. [file TSWJ-2026-7661411-s001.docx]

**Supplementary FIGURE 1:** Phytochemical Structure of SAL-ME Compounds.
